# Impact of pre-treatment with melatonin on hematological alterations and splenic lipid peroxidation in phenylhydrazine-induced acute hemolytic anemia in Wistar rats

**DOI:** 10.1016/j.htct.2026.108546

**Published:** 2026-10-31

**Authors:** Vitória Rosa de Oliveira, Custódio José Gaspar, Silvio Tasca, Elissa Kerli Fernandes, Cristina Campos Carraro, Adriane Belló-Klein, Fernanda Visoli, Alexandre Luz de Castro, Alex Sander da Rosa Araujo

**Affiliations:** a Laboratory of Cardiovascular Physiology, Physiology Department, Institute of Basic Health Science, Federal University of Rio Grande do Sul (UFRGS), R. Ramiro Barcelos, 2600 - Santa Cecília, Porto Alegre, RS, 90035-003, Brazil; b Federal University of Rio Grande do Sul (UFRGS), Oral Pathology Laboratory of the Faculty of Dentistry, Porto Alegre, Brazil

**Keywords:** Anemia, Phenylhydrazine, Oxidative stress, Redox balance, Antioxidants, Melatonin, Hemolysis

## Abstract

**Introduction:**

Hemolytic anemias are characterized by the premature destruction of red blood cells, leading to redox imbalance in the organism. Under these conditions, organs such as the spleen may be particularly affected. Melatonin is known for its anti-inflammatory and antioxidant properties and has emerged as a promising therapeutic candidate. Thus, the present study investigated the impact of melatonin pretreatment in male Wistar rats subjected to phenylhydrazine-induced acute hemolytic anemia.

**Methods:**

Twenty-eight male Wistar rats were randomly assigned to four experimental groups: control, anemic and untreated, non-anemic treated with melatonin, and anemic treated with melatonin. Hematological analyses were performed using whole blood samples, while spleen tissue was used for biochemical assessments of oxidative stress and morphometric analyses.

**Results:**

The results showed a negative correlation between thiobarbituric acid-reactive substances and hemoglobin levels (r = −0.8406; p < 0.0001). Hemolysis also induced an antioxidant counterregulatory response, evidenced by increased thiol levels (p = 0.0005). Moreover, sulfhydryl levels were positively correlated with thiobarbituric acid-reactive substance levels (r = 0.8108; p < 0.0001). Nevertheless, melatonin did not restore hematological parameters in anemic animals and did not prevent the increase in splenic lipid peroxidation.

**Conclusion:**

Splenic tissue exhibited an antioxidant response to oxidative damage induced by hemolysis, and lipid peroxidation was associated with the severity of anemia. However, under the conditions tested, melatonin was ineffective in attenuating oxidative stress or hematological alterations associated with acute hemolytic anemia.

## Introduction

Anemia comprises a group of diseases characterized by reduced red blood cell counts, manifesting as decreased hemoglobin and hematocrit levels and often accompanied by changes in red blood cell morphology. This condition compromises the adequate oxygen supply to tissues, generating symptoms such as fatigue, pallor, drowsiness, and tachycardia [1]. Approximately 30% of the world’s population has some type of anemia, reinforcing its relevance as a global public health problem. In this context, hemolytic anemias are characterized by the early and severe destruction of erythrocytes, whose average physiological lifespan is approximately 120 days [2].

The hemolytic process can occur intravascularly through the lysis of erythrocytes directly in the vascular lumen. Furthermore, extravascular hemolysis occurs in the spleen due to erythrocyte senescence or altered deformability, a process mediated by the reticuloendothelial system [3]. Genetic alterations are the main cause of congenital anemias, such as deficiency of the enzyme glucose-6-phosphate dehydrogenase (G6PD) [4]. G6PD deficiency causes a reduction in the production of nicotinamide adenine dinucleotide phosphate (NADPH), a key molecule in the recycling of glutathione. As a result, erythrocytes become more susceptible to hemolysis due to oxidative stress [5]. The experimental PHZ model stimulates oxidative processes, generating a cellular environment with characteristics similar to those observed in G6PD deficiency [6].

In this context, an aerobic environment enables the formation of reactive oxygen species (ROS), chemical species that play key roles in the organism [7]. Likewise, reactive nitrogen species (RNS) also appear to be important in cellular physiology. However, although ROS and RNS have recognized physiological roles, high concentrations of these species in cells can trigger oxidative and nitrosative stress. In this sense, both ROS and RNS tend to interact with various cellular structures, such as nucleic acids and membrane lipids, while also contributing to protein modifications and possible changes in apoptosis and cell proliferation pathways in different tissues of the organism [8]. Therefore, a robust antioxidant system is important for maintaining appropriate levels of these species.

Melatonin (N-acetyl-5-methoxytryptamine), the main hormone synthesized by the pineal gland, is also produced at extrapineal sites, such as the gastrointestinal tract, skin, and mitochondria, and is known for its potent antioxidant and anti-inflammatory properties [9]. Its ability to cross cell membranes, as well as to donate electrons and directly stabilize free radicals, gives this molecule a high antioxidant capacity [10]. Melatonin and its active metabolites, 6-hydroxymelatonin [6(OH)M], N1-acetyl-N2-formyl-5-methoxykynuramine (AFMK), and 5-methoxytryptamine, also act via G-protein-coupled melatonin type 1 and 2 receptors (MT1 and MT2), as well as nuclear aryl hydrocarbon receptors (AhR) and peroxisome proliferator-activated receptor γ (PPARγ), to modulate protective cellular pathways [11].

The spleen is an important secondary lymphoid organ that performs fundamental hematopoietic and immunological functions and is responsible for extravascular hemolysis [12]. The red pulp of the spleen is responsible for selecting and eliminating senescent erythrocytes, or red blood cells with altered cellular structure, through the reticuloendothelial macrophage system. In the context of hemolytic anemia, there may be an increase in heme and iron concentrations, leading to increased ROS production in splenic tissue [13]. Consequently, the disruption of redox homeostasis induced by hemolytic anemia in the spleen could compromise not only its hematological function but also its role in immune regulation. However, there are limited data in the literature exploring the antioxidant potential of melatonin in the spleen under conditions of hemolytic anemia and its impact on splenic function. Therefore, the present study investigated the antioxidant role of melatonin in the spleen of male Wistar rats subjected to acute phenylhydrazine (PHZ)-induced hemolytic anemia through the evaluation of hematological parameters and markers of oxidative stress.

## Materials and methods

Male Wistar rats (*Rattus norvegicus*) (N = 28), 21 days old and weighing approximately 45 g, from the Center for Reproduction and Experimentation of Laboratory Animals (CREAL) of the Federal University of Rio Grande do Sul (UFRGS) were used. The animals were housed in the Sectoral Animal Facility of the Department of Pharmacology, Institute of Basic Health Sciences (ICBS), at the same university. The animals were kept in polypropylene cages with sawdust-covered floors, containing two to four animals per cage. The housing environment had a temperature of 22 °C (± 2 °C), a 12-hour light-dark cycle, and a relative humidity of 40%–60%. The animals had free access to water and food (ad libitum). Prior to the start of the experimental procedures, the animals underwent a 14-day acclimation period to the new environment and to the researchers responsible for the study. All interventions and experimental techniques were performed by qualified research personnel. All procedures in this study were conducted in accordance with the State Animal Protection Code of Rio Grande do Sul, the guidelines of the National Council for the Control of Animal Experimentation (CONCEA), and the International Guiding Principles for Biomedical Research Involving Animals established by the Council for International Organizations of Medical Sciences (CIOMS). This project was evaluated and approved by the Ethics Committee on the Use of Animals (CEUA) of UFRGS under Approval Letter Nº 45.707.

### *Sample size calculation*

Sample size calculation was performed using Microsoft Excel Home and Student 2019. The following parameters were considered: probability of error α = 0.05 and statistical power (1 − β) = 0.80, using experimental oxidative stress data to obtain the required statistical parameters (means, standard deviations, and sample size) [14]. Based on these calculations, the required sample size was 22 animals. However, because of the mortality rate inherent to the experimental model, an additional 25% was added to the total number of rats, resulting in a final sample size of 28 animals, which were randomly divided into four groups.

### *Experimental design*

The animals were numbered from 1 to 28, and randomization was performed using Microsoft Excel 2019, which randomly allocated them to their respective experimental groups. The experimental groups were established as follows: Control (CTRL), n = 7; Anemia (PHZ), n = 7; Melatonin (MEL), n = 7; and Melatonin + Anemia (MEL+PHZ), n = 7. To investigate the potential protective role of melatonin in PHZ-induced acute hemolytic anemia, melatonin solution was administered orally by gavage at a dose of 20 mg/kg as a pretreatment for 14 days [14]. Subsequently, the acute hemolytic anemia model was induced by intraperitoneal administration of up to two doses of PHZ (40 mg/kg each), with a 24-hour interval between administrations [15]. For both melatonin and PHZ treatments, 10% dimethyl sulfoxide (DMSO) solution and distilled water was used as the vehicle, respectively, and the control group received the vehicle alone. The animals were euthanized 24 h after the final intraperitoneal administration of either PHZ solution or distilled water, according to the CONCEA Guidelines for the Practice of Euthanasia.

### *Sample collection and storage*

Blood was immediately collected into tubes containing ethylenediaminetetraacetic acid as an anticoagulant and was used for hematological analysis on the same day of collection. After blood collection, a blood smear was prepared using May-Grunwald-Giemsa staining. Both red (erythrocyte morphology, presence of polychromasia, presence of erythroblasts) and white (morphology and leukocyte differential) cell series were qualitatively evaluated. An additional blood fraction was collected in a tube containing a clot activator to obtain serum for subsequent biochemical analyses. The spleen was collected and weighed immediately after euthanasia. Following collection, the organ was washed in 0.9% sodium chloride solution. The spleen and blood serum samples were then stored at −20 °C until biochemical analyses were performed.

### *Estimation of the establishment of experimental anemia*

The hemoglobin and indirect bilirubin levels, as well as erythrocyte morphology, presence of polychromasia, presence of erythroblasts, Howell–Jolly bodies, and reticulocytosis were used to estimate the experimental hemolytic anemia level [16]. These evaluations were performed at the end of the experimental protocol. After euthanasia, blood was collected and hemoglobin and total and indirect bilirubin levels were measured. Based on this criterion, two animals were excluded from the statistical analyses, one from the PHZ group and one from the MEL+PHZ group. In addition, five animals died during the induction of hemolytic anemia with PHZ. Therefore, the final sample size used in this study was 21 animals (CTRL: n = 5; PHZ: n = 5; MEL: n = 6; and MEL+PHZ: n = 5).

### *Hematological analyses*

Hematological analyses were performed at the Veterinary Clinical Analysis Laboratory (LACVET) of the School of Veterinary Medicine, Federal University of Rio Grande do Sul (UFRGS). The following parameters of the erythrocyte series were evaluated: erythrocyte count, hemoglobin concentration, red blood cell distribution width (RDW), mean corpuscular volume (MCV), and mean corpuscular hemoglobin (MCH). Total and differential leukocyte counts were also determined. These parameters were measured using an automated method with the ProCyte Dx Hematology Analyzer (Westbrook, ME, USA).

### *Reticulocyte count*

A manual reticulocyte count was performed using the brilliant cresyl blue staining method. After incubation at 37 °C, blood smears were prepared using the wedge-smear technique and analyzed by light microscopy. The final value was expressed as the percentage (%) of reticulocytes, according to the following equation: percentage (%) of reticulocytes = (number of reticulocytes counted × 100) / 1000.

### *Total and indirect bilirubin levels*

Serum total bilirubin levels were measured using a method based on the diazotization reaction (diazonium salt) and expressed in milligrams per deciliter (mg/dL). This evaluation was performed using a commercial kit from the Alinity c line (Abbott Core Laboratory).

### *Splenomegaly analysis*

The splenomegaly index was calculated as the ratio between spleen weight (g) and body weight (g).

### *Homogenization and protein quantification of samples*

Approximately 100 mg of tissue was used to prepare the homogenate. A solution containing 1.15% potassium chloride and 1% phenylmethanesulfonyl fluoride was added at a dilution ratio of 1:10 (w/v) for spleen samples. Homogenization was performed using a tissue homogenizer with a 40-second pulse. After homogenization, the samples were centrifuged at 1000 × *g* for 20 min at 4 °C. The resulting supernatant was collected and immediately stored at −20 °C for subsequent biochemical analyses. A portion of the supernatant from each homogenate was used for total protein quantification using the Lowry method [17].

### *Nitrite analysis*

Nitrite levels were assessed using the Griess colorimetric method. This assay is based on the formation of a colored compound resulting from the reaction of nitrite present in the sample with Griess reagent (1% sulfanilamide and 0.1% naphthylethylenediamine) under acidic conditions [18]. The results were expressed as millimoles of nitrite (mM NO₂⁻).

### *Analysis of total ROS*

Total ROS levels were determined using a method based on the oxidation of 2′,7′-dichlorodihydrofluorescein diacetate (DCFH-DA) to 2′,7′-dichlorofluorescein (DCF), a highly fluorescent compound generated in the presence of intracellular ROS [19]. The results were expressed as picomoles per milligram of protein (pmol/mg protein).

### *Sulfhydryl analysis*

Sulfhydryl levels were determined using a colorimetric method based on the reaction of 5,5′-dithiobis(2-nitrobenzoic acid) (DTNB) with thiol groups present in the samples [20]. Results were expressed as nanomoles of TNB per milligram of protein (nmol TNB/mg protein).

### *Lipid peroxidation analysis*

Lipid peroxidation in the samples was assessed by quantifying the reaction of oxidation products with thiobarbituric acid-reactive substances (TBARS) [21]. The results were expressed as nanomoles per milligram of protein (nmol/mg protein).

### Statistical analysis

The data obtained were analyzed using GraphPad Prism 8.0.2 (Windows). Initially, the data were subjected to the Shapiro–Wilk test to assess normality. Data with a normal distribution were analyzed using one-way analysis of variance, followed by Bonferroni's post hoc test for multiple comparisons among the experimental groups. Two-way analysis of variance and Pearson’s correlation analysis were also used for specific evaluations. Results were considered statistically significant at p < 0.05.

## Results

### *Hematological analyses demonstrate the establishment of phenylhydrazine-induced hemolytic anemia*

The hematological analyses demonstrated that the hemolytic anemia model was successfully established, as the main hematological parameters associated with this condition showed changes consistent with acute hemolytic anemia in the animals that received PHZ. Considering the hemoglobin and erythrocyte values (Figure 1a and b, respectively), the PHZ and MEL+PHZ groups showed a significant reduction compared with the CTRL and MEL groups (p <0.0001). By contrast, the RDW and reticulocyte values (Fig. 1, Fig. 1, respectively) were higher in the PHZ and MEL+PHZ groups relative to the CTRL and MEL groups (p <0.05). The MCV values did not differ among the experimental groups. However, the PHZ and MEL+PHZ groups showed increased MCH values compared with the control groups (p <0.05) (Fig. 1, Fig. 1). Regarding total and indirect bilirubin levels, the PHZ and MEL+PHZ groups showed significantly higher values than the CTRL and MEL groups (p <0.05), whereas no differences were observed between the PHZ and MEL+PHZ groups or between the CTRL and MEL groups (Fig. 1, Fig. 1). Moreover, melatonin administration at the dose used had no significant effect on any of the hematological parameters evaluated in either the MEL or MEL+PHZ groups. The total leukocyte count was increased in the PHZ and MEL+PHZ groups compared with the CTRL and MEL groups (p <0.05) (Figure 1i). In addition, the PHZ and MEL+PHZ groups showed an approximately 12-fold increase in segmented neutrophil counts compared with the CTRL group (Figure 1j).

**Fig. 1:**
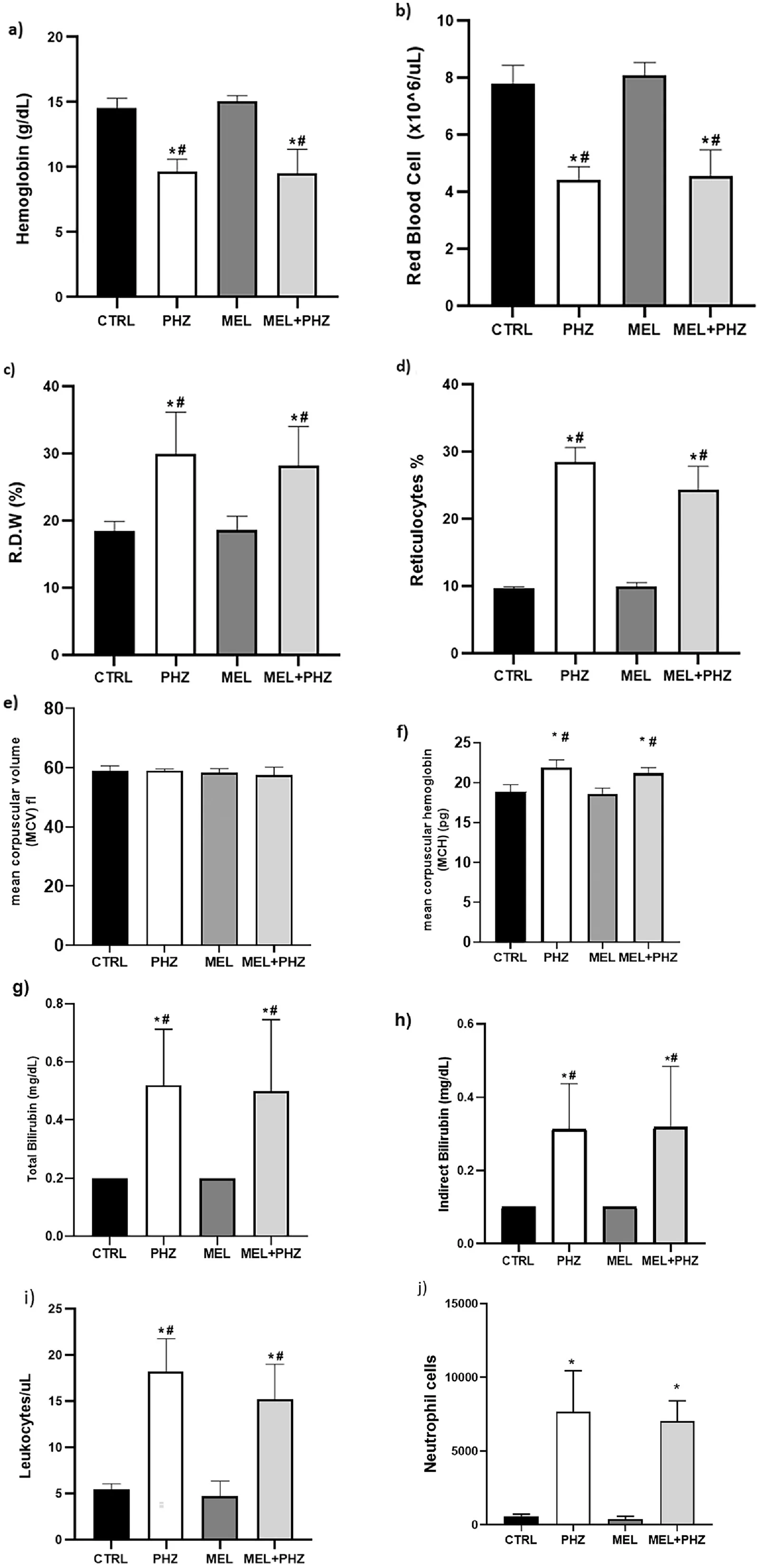
Results obtained through hematological analyses of the four experimental groups (Control [CTRL]: n = 5; Anemia [PHZ]: n = 5; Melatonin [MEL]: n = 6; and Melatonin + Anemia [MEL+PHZ]: n = 5). Results were evaluated using one-way ANOVA followed by the Bonferroni post hoc test for multiple comparisons. (a) Hemoglobin values (g/dL). (b) Erythrocyte values (x 10^6^/µL). (c) Erythrocyte distribution width (RDW) (%), (d) reticulocyte percentage, (e) Mean corpuscular volume (MCV), (f) Mean corpuscular hemoglobin (MCH), (g) total bilirubin levels, (h) indirect bilirubin levels (i) total leucocyte count, and (j) differential leucocyte count. [*] Statistically significant difference in relation to the CTRL group. [#] Statistically significant difference in relation to the MEL group.

### *Hematological changes observed in blood smear slides: evidence of hemolytic anemia*

Analysis of blood smear slides from the different experimental groups showed that the CTRL and MEL groups (Figure 2a and b) presented erythrocytes with normocytic and normochromic characteristics, as well as a low number of reticulocytes in peripheral circulation. By contrast, the PHZ and MEL+PHZ groups exhibited a reduction in erythrocyte numbers, associated with marked anisocytosis, polychromasia, and reticulocytosis (Fig. 2, Fig. 2). Howell–Jolly bodies were also identified within erythrocytes in the PHZ and MEL+PHZ groups.

**Fig. 2:**
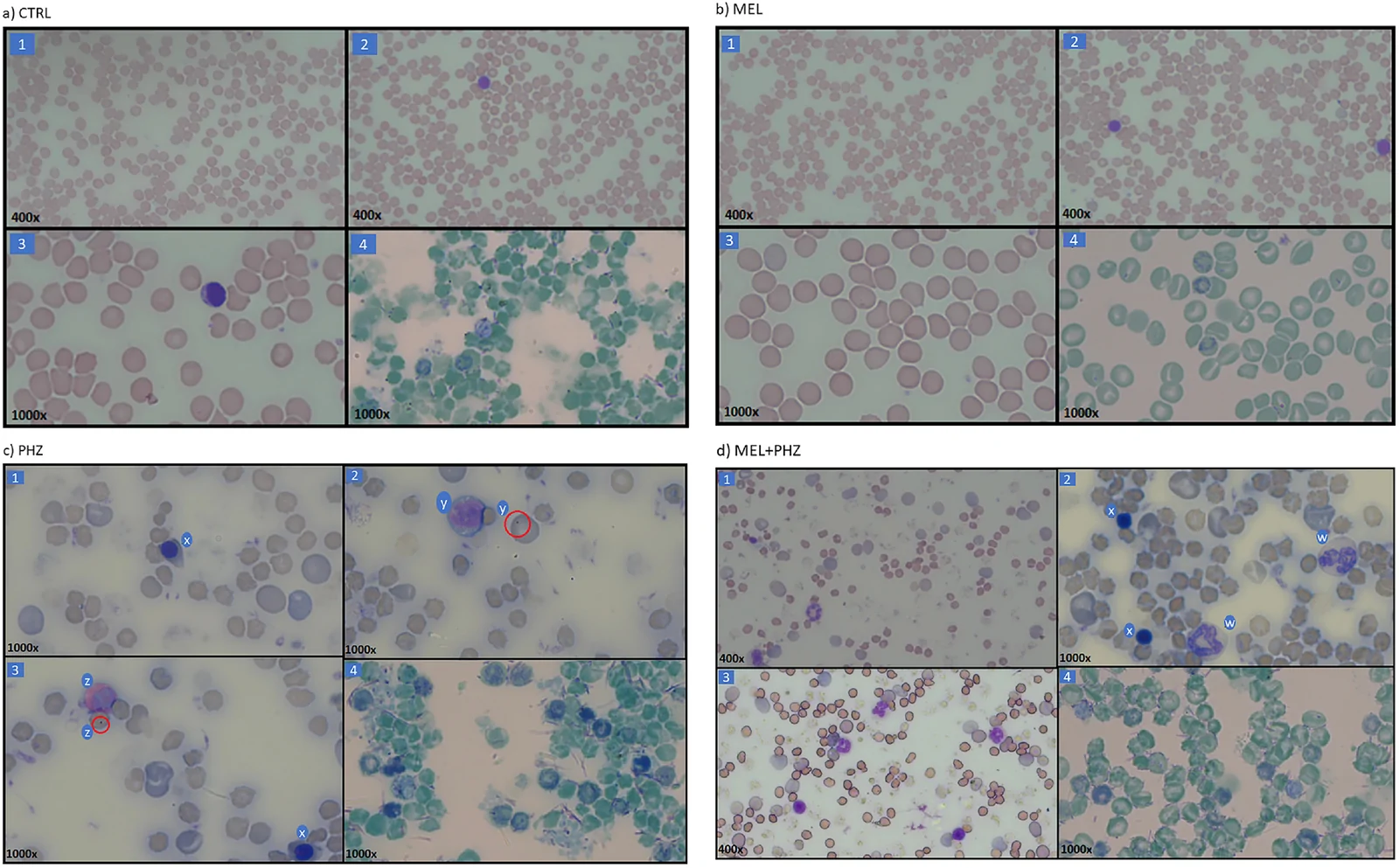
Blood smears from animals across experimental groups: (a) Control (CTRL) group (b) Anemia (PHZ) group (c) Melatonin (MEL) group and Melatonin + Anemia (MEL+PHZ) group. Panels (1), (2), and (3) are stained with May-Grünwald-Giemsa stain, highlighting lymphocytes in (2) and (3). Panel (4) is stained with brilliant cresyl blue, highlighting reticulocytes. (x) erythroblastosis, (y) atypical cell and Howell-Jolly corpuscles [identified by red circle], (z) eosinophil and Howell-Jolly corpuscles [identified by red circle]. (w) neutrophils.

### *Splenic morphological analysis*

The PHZ and MEL+PHZ groups showed significantly increased spleen weight and size (cm) compared with the CTRL and MEL groups (p <0.0005) (Figure 3a and b). However, no significant differences in spleen weight were observed between the PHZ and MEL+PHZ groups.

**Fig. 3:**
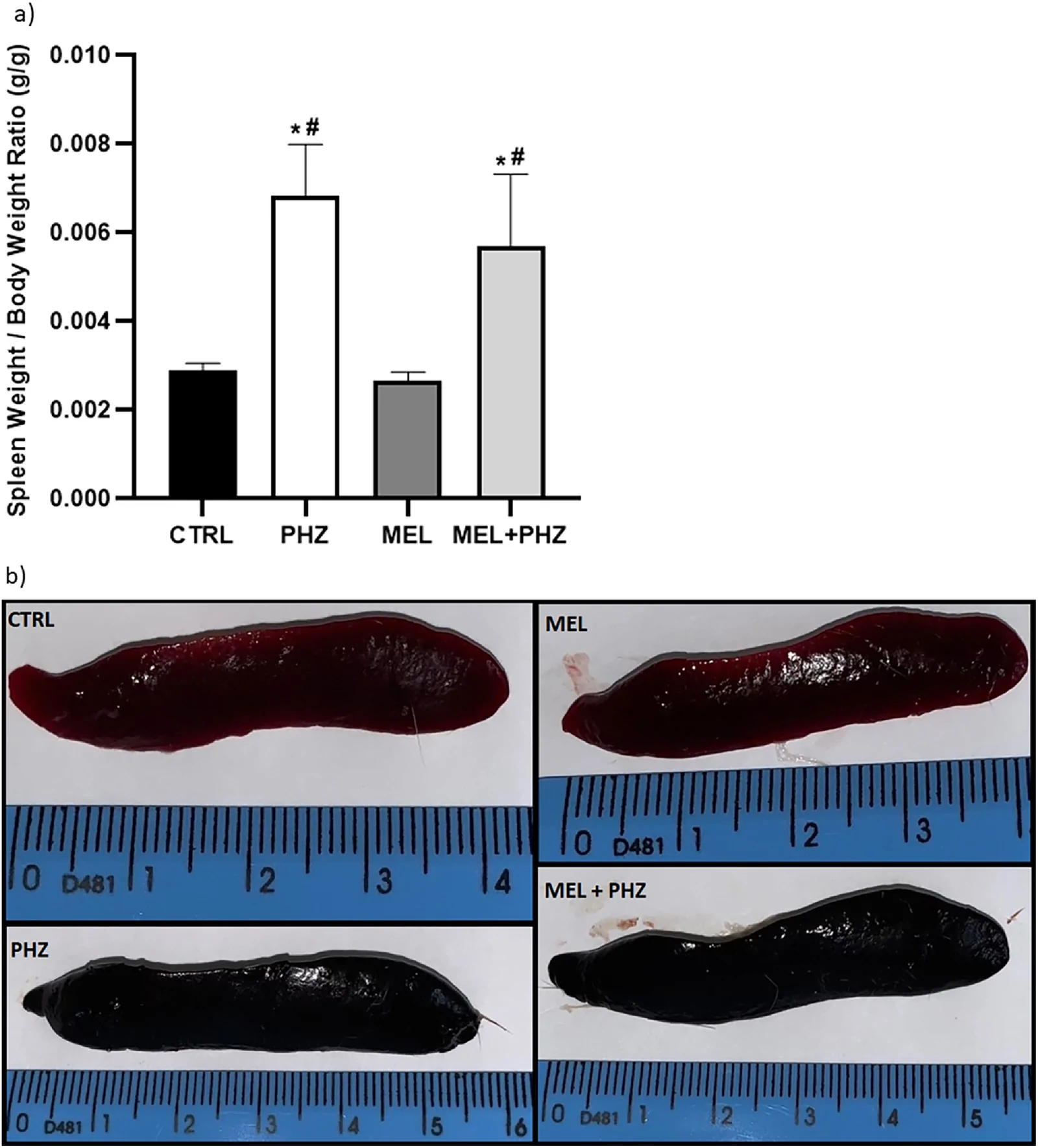
a) Ratio between spleen weight (g) and body weight (g) of animals in different experimental groups (Control [CTRL]; Anemia [PHZ]; Melatonin [MEL]; and Melatonin + Anemia [MEL+PHZ]). b) Macroscopic representation of spleens collected from animals in the different experimental groups. Note the different amplitudes in the image scale, demonstrated by the blue ruler. The variation in splenic size (cm) between groups is evident, with emphasis on its increase in the PHZ and MEL + PHZ groups compared to the CTRL and MEL groups. Results were evaluated using one-way ANOVA followed by the Bonferroni post hoc test for multiple comparisons. [*] Statistically significant difference in relation to the CTRL group. [#] Statistically significant difference in relation to the MEL group.

### *Impact of melatonin pretreatment on oxidative damage in the spleen*

The PHZ and MEL+PHZ groups exhibited increased nitrite and sulfhydryl levels compared with the CTRL and MEL groups (Figure 4a and b). However, ROS levels were decreased in the PHZ and MEL+PHZ groups relative to the CTRL and MEL groups (p <0.05) (Figure 4c). In addition, lipid peroxidation levels (TBARS) were increased in the PHZ and MEL+PHZ groups compared with the CTRL and MEL groups (p <0.05) (Figure 4d). Correlation analysis revealed a positive association between sulfhydryl group levels and TBARS levels (r = 0.8108; p <0.0001) (Figure 5a).

**Fig. 4:**
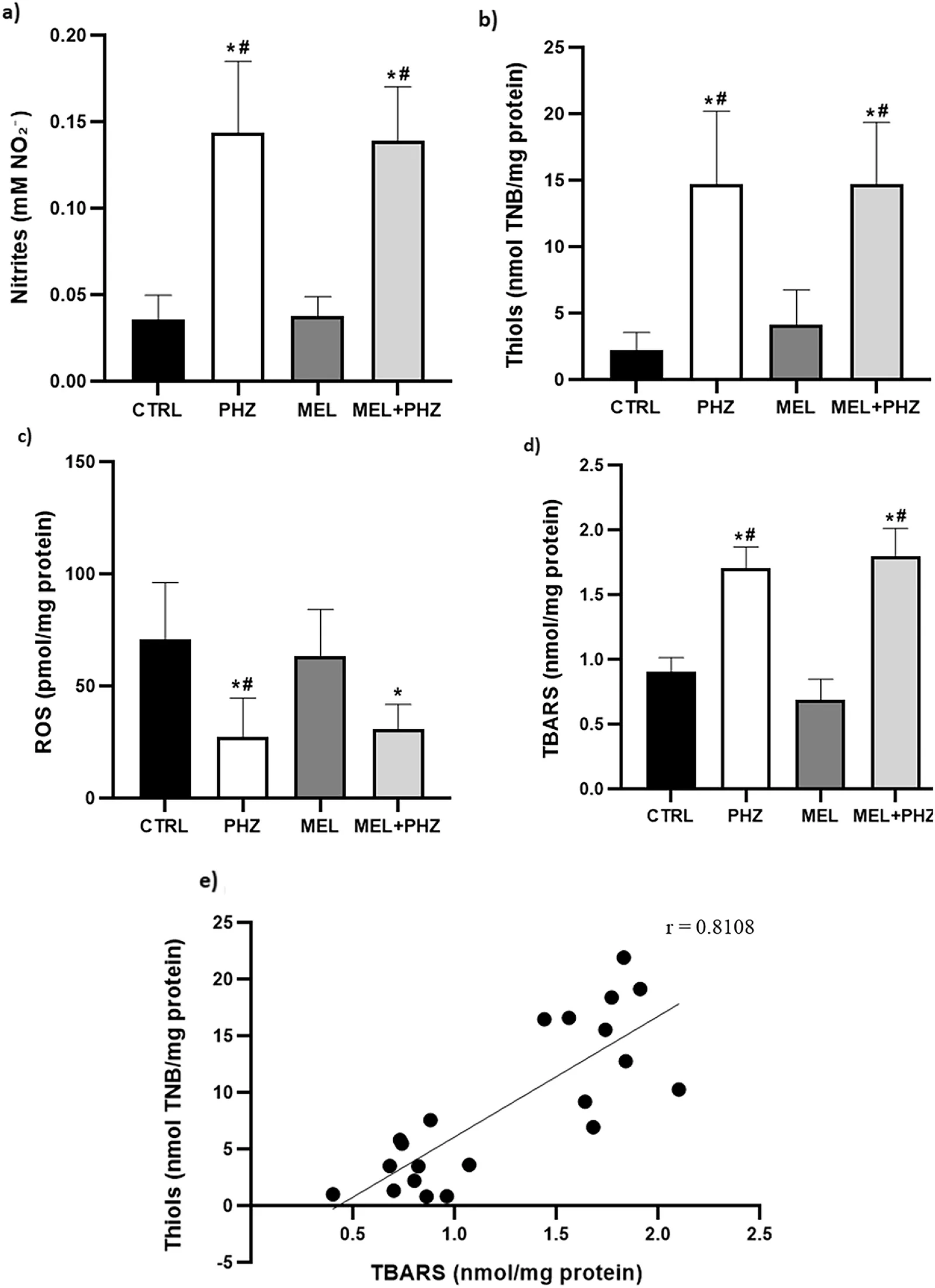
Results obtained in the biochemical analysis of the spleen. Results were evaluated using one-way ANOVA followed by the Bonferroni post hoc test for multiple comparisons. (a) Nitrites (mM NO₂⁻). (b) Thiol Groups (nmol TNB/mg of protein). (c) Total Reactive Oxygen Species (ROS) (pmol/mg of protein). (d) TBARS (nmol/mg of protein). (Control [CTRL]; Anemia [PHZ]; Melatonin [MEL]; and Melatonin + Anemia [MEL+PHZ]).[*] statistical difference in relation to the CRTL group.[#] statistical difference in relation to the MEL group.

**Fig. 5:**
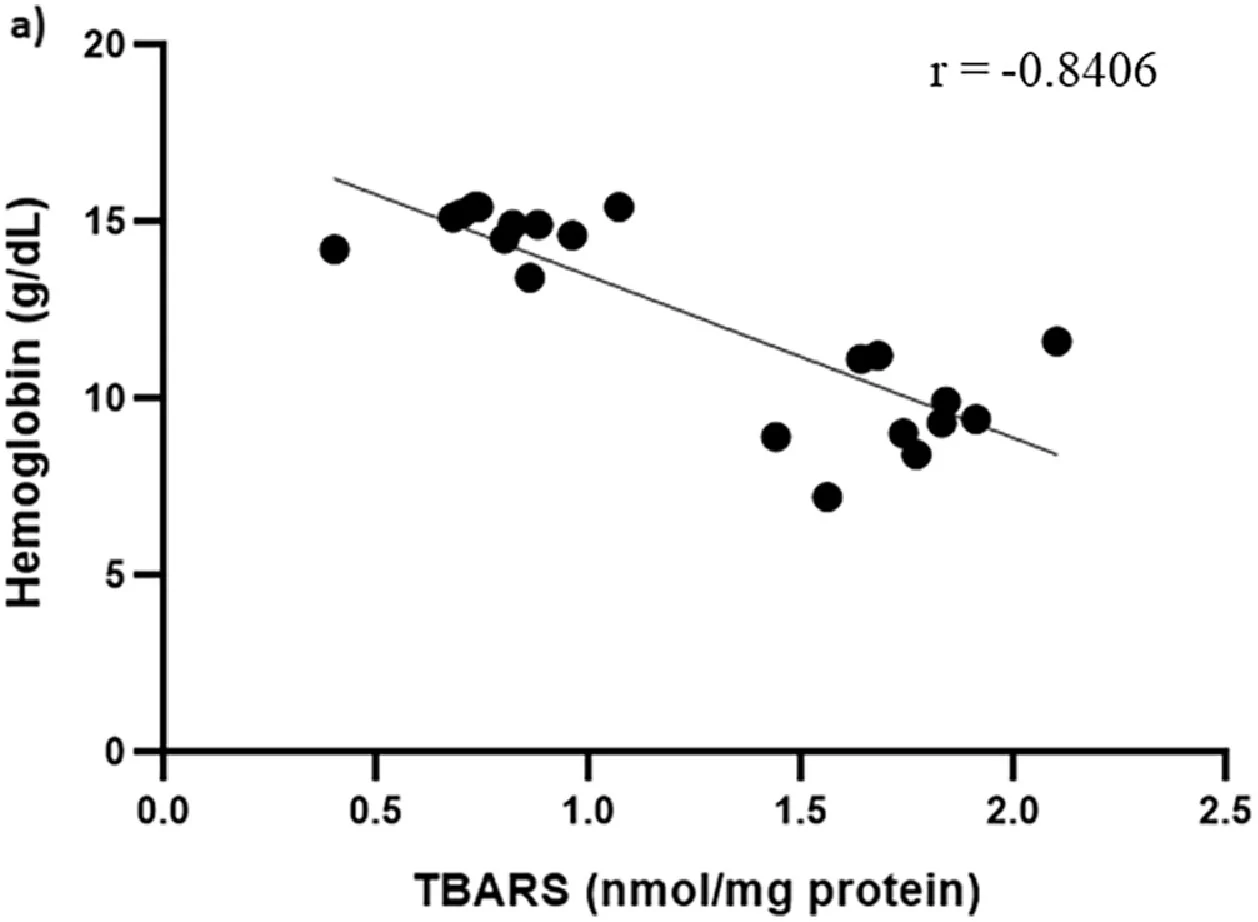
Results obtained by Pearson’s correlation a) Values of Thiol Groups in relation to the Lipid Peroxidation levels in the spleen. b) Hemoglobin concentration in relation of lipid peroxidation levels in the spleen.

### *Possible impact of splenic lipid peroxidation on hematological indices*

A significant negative correlation was observed between hemoglobin levels and lipid peroxidation (r = −0.8406; p <0.0001) (Figure 5b).

## Discussion

The present study was performed to investigate the impact of acute hemolytic anemia on splenic oxidative stress. Furthermore, it investigated whether pretreatment with melatonin could serve as a protective factor against lipid peroxidation. In this study, acute hemolytic anemia was associated with reticulocytosis, leukocytosis, increased TBARS levels, and activation of a non-enzymatic antioxidant counterregulatory response (increased thiol levels) in splenic tissue. Moreover, the data suggest an association between hemolysis and lipid peroxidation. However, under these experimental conditions (dose and duration of treatment), melatonin did not mitigate the oxidative stress or hematological alterations induced by acute hemolytic anemia.

The results suggest changes compatible with hemolytic anemia in the groups receiving PHZ (PHZ and MEL+PHZ). Important cellular findings, characteristic of hemolytic anemia, were observed in the microscopic analyses of the PHZ and MEL+PHZ groups. A high degree of anisocytosis and polychromasia, as well as the presence of platelet aggregates, Howell–Jolly bodies, ghost erythrocytes, and cellular fragments, were among these findings. Furthermore, increased numbers of reticulocytes were observed in the peripheral circulation of the PHZ and MEL+PHZ groups, which may indicate bone marrow hyperreactivity in response to hemolysis induced by acute hemolytic anemia [22]. Elevated MCH values may also be associated with the increased reticulocyte count commonly observed in hemolytic anemias. In addition, the increased total and indirect bilirubin levels in the PHZ and MEL+PHZ groups suggest substantial hemolysis. The increased total leukocyte and neutrophil counts also appear to be associated with the inflammatory response induced by hemolytic anemia. However, leukocyte evaluations alone are not sufficient to determine the inflammatory consequences of hemolysis. Taken together, these findings suggest that the hemolytic anemia induction model was successfully established in the present experimental protocol. Melatonin had no observed effect on the evaluated hematological parameters. By contrast, Ajibade et al. reported that melatonin treatment improved hemoglobin levels in a model of hemolytic anemia [6]. This discrepancy may be related to differences in experimental design, particularly with respect to the dose and duration of melatonin treatment.

The spleen, a secondary lymphoid organ, is highly specialized at identifying aged or defective blood cells, as well as detecting microorganisms and other antigens [23]. In this study, acute hemolytic anemia promoted splenic hypertrophy in animals of the PHZ and MEL+PHZ groups compared with the CTRL and MEL groups. Furthermore, splenomegaly was accompanied by a dark red coloration of the spleens in PHZ-treated animals, a condition consistent with the pathophysiological pattern observed in hemolytic anemia [24,25]. In this context, melatonin did not appear to reverse the splenic congestion caused by exacerbated hemolysis because no significant differences were observed between the PHZ and MEL+PHZ groups. The spleen contains specialized cells essential for this function, particularly red pulp macrophages, which are responsible for the processing and elimination of heme and iron released during erythrocyte degradation [26]. In this context, these hemolytic products may disrupt redox homeostasis and contribute to oxidative stress [27].

Thus, the current results showed that PHZ and MEL+PHZ groups presented increased markers of oxidative damage in the spleen, such as lipid peroxidation. Conversely, sulfhydryl and nitric oxide levels increased significantly in both the PHZ and MEL+PHZ groups. This elevation suggests a potential counterregulatory response to acute hemolytic anemia-induced oxidative stress, which may account for the reduced ROS levels observed in these groups [28]. Melatonin does not appear to directly interfere with these oxidative stress parameters. Nevertheless, melatonin and its metabolites are known to exert antioxidant effects through free radical scavenging, thereby reducing ROS bioavailability and oxidative stress [29]. In addition, melatonin can interact with MT1 and MT2 receptors and activate intracellular signaling pathways that regulate transcription factors such as nuclear factor erythroid 2-related factor 2 (NRF2), promoting the synthesis of antioxidant enzymes and enhancing cellular protection against disruptions in redox homeostasis [29]. Previous studies have demonstrated a potential protective effect of melatonin in hemolytic anemia [30,31]. In 2006, Erat & Ciftci reported a positive effect on glutathione reductase activity in erythrocytes [30]. Moreover, in 2005, Piriyakhuntorn et al. demonstrated that patients with thalassemia who received melatonin (20 mg/day for one year) exhibited decreased lipid peroxidation levels [31]. Differences in the anemia models used (in the dose and duration of melatonin administration) may explain the discrepancy between the current findings and those reported in the literature.

## Study limitations

Our protocol of hemolytic anemia determination uses indirect bilirubin and hemoglobin concentrations; however, there are other more specific approaches that could be used, such as plasma heme, lactate dehydrogenase, or haptoglobin. Moreover, this study would benefit from an experimental design evaluating melatonin administration at different doses and treatment durations. Likewise, assessment of antioxidant enzyme activity could provide additional information regarding oxidative stress and contribute to a more robust evaluation of the antioxidant effects of melatonin. Even though this study has shown inflammatory cellular components, these parameters are not enough. In this sense, another analysis on effects of melatonin and hemolysis on inflammatory pathways would strengthen the study. Finally, because melatonin was administered orally, an assessment of its bioavailability could provide valuable information for future investigations.

## Conclusions

In the present study, PHZ-induced acute hemolytic anemia in Wistar rats was associated with marked hematological impairment, splenomegaly, increased splenic lipid peroxidation, and elevated thiol and nitrite levels. However, melatonin pretreatment at 20 mg/kg for 14 days did not ameliorate these alterations. These findings are limited by the specific conditions of the experimental protocol used for both the melatonin and PHZ interventions. Therefore, future studies should explore different doses, treatment regimens, and mechanistic endpoints of melatonin treatment in this anemia hemolytic model.

## Data availability

The data that support the findings of this study are available from the corresponding author upon reasonable request.

## Conflicts of interest

The authors declare no competing interests.
